# Formation of Condyle-Like Structure after Treatment of Temporomandibular Joint Ankylosis: Literature Review and Long-Term Follow-Up of Two Patients

**DOI:** 10.1155/2017/9060174

**Published:** 2017-10-02

**Authors:** Orhan Güven

**Affiliations:** Department of Oral and Maxillofacial Surgery, Faculty of Dentistry, Ankara University, Ankara, Turkey

## Abstract

Treatment of ankylosis is one of the greatest challenges in temporomandibular joint (TMJ) surgery. To provide a satisfactory mouth opening, as well as normal jaw function, and to prevent reankylosis in the long term are the most important principles in the treatment of TMJ ankylosis. These functions have been attained in both of the presented patients in the long term. It is known that heterotopic bone formation is rare in the maxillofacial area, but rapid bone regeneration which reconstitutes a new condyle is rarer. The purpose of the presented paper is to reveal the existence of an inherent capability of the mandible, rapid bone growth of the ramus mandible, and reformation of a previously nonexisting condyle after resection of the ramus in patients with TMJ ankylosis. In this paper, two unusual cases of unexpected condyle-like structure formation after treatment of ankylosis were presented.

## 1. Introduction

Temporomandibular joint (TMJ) ankylosis is an affliction which occasions much misery for the unfortunate victim. In early childhood, it may give rise to facial asymmetry and the life of the patient may, at any moment, be jeopardized by transient or trivial obstruction of the airway; the very nature of the diet which has perforce to be consumed predisposes towards the development of caries and periodontal problems. The most common etiology of ankylosis is trauma in adults and trauma and infection in children [1–3].

Treatment of ankylosis is probably one of the greatest challenges in TMJ surgery. Timing, the type of operation, and the protocol of treatment vary from one country to another. However, the main principles include resection of the ankylosed segment, use of interposition materials, and postoperative physiotherapy [4–6].

The vast number of techniques tried over the years illustrates the difficulty that has been experienced in obtaining a satisfactory method for the treatment of TMJ ankylosis. First, gap arthroplasty was described and commonly used; then, interposition materials were inserted later to prevent reankylosis. A variety of autologous grafts, such as fascia, muscle, dermis, cartilage, and bone, have been tried over the years [7–10]. In time, some alloplastic materials such as acrylic, silicone rubber, gold foil, and tantalum foil have been used for the same purpose [11–14]. Following the improvements in joint prosthesis in orthopedic surgery, coated or noncoated hemijoint and total joint prosthesis were developed for the reconstruction of the TMJ [15–20].

Variable success rates for a variety of interposition materials and techniques have been reported in the treatment of TMJ ankylosis with limited follow-up periods.

Gap arthroplasty is the simplest approach described to disconnect the ascending part of the mandible from the skull base. The possibility of heterotopic bone formation is too high in this technique. Following resection, the gap is inserted into network capillaries. This process of revascularization is mainly derived from the remaining bone stump and surrounding host tissues. The osteogenic cells which are derived from capillaries subsequently act as foci of new bone formation [21, 22].

Fascia [10] and silastic [23] were used to prevent reankylosis. The difficulty lied in the fixation and in keeping these interposition materials in a proper position during function.

Autogenous bone replacement for the treatment of ankylosis has been proposed as an alternative technique [1, 19]. Bone grafts serve different functions. These include osteogenesis and osteoinduction. On the other hand, when used as a reconstruction material in TMJ, how this bone graft prevents itself from coming into contact with osteogenic cells which are derived from capillaries of surrounding host tissues and formation of heterotopic bone has never been explained.

Alloplastic total TMJ reconstruction is regarded as an entirely biomechanical rather than a biologic solution for severe anatomic diseases. Total joint reconstruction should be considered as a management option for patients with severe anatomically, iatrogenically, or pathologically compromised, dysfunctional TMJs. Besides the other disadvantages, alloplastic TMJ implant never follows the patient's growth [18].

The metallic fossa prosthesis acts as an interposition material to eliminate bone-on-bone contact in patients with osteoarthritis and thereby to prevent reossification of bone in TMJ ankylosis [20, 24].

What happens to the resected segments in the long term after gap arthroplasty or with use of a spacer, autogenous bone graft, hemijoint, and total joint prosthesis has never been explained so far.

Attaining spontaneous healing and rapid bone regeneration by functional treatment after condyle fractures in growing patients is a well known clinical condition [2, 25, 26]. On the other hand, spontaneous bone regeneration in growing patients and in adults after resection of the large proportions of the mandible has been rarely reported by maxillofacial surgeons. At the beginning, authors reported this phenomenon as “unusual rapid bone regeneration following mandibular resection” [27–32]. In the following years, some other studies followed, speculating on reasons of the new bone formation after partial removal of the mandible [33–37]. The purpose of the presented paper is to reveal the existence of an inherent capability of the mandible, rapid bone growth of the ramus mandible, and formation of a condyle-like process after resection of the ramus for the treatment of TMJ ankylosis in two patients.

## 2. Report of Cases

### 2.1. Case  1

**Figure 1:**
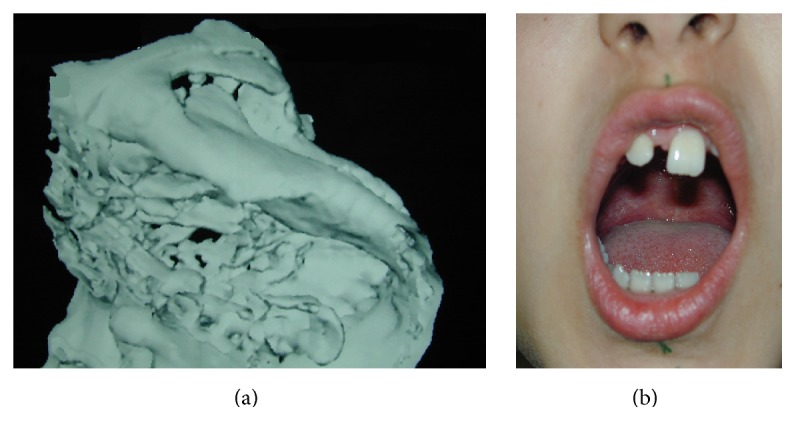
(a) Ankylosis of the right TMJ during the patient's (Case  1) first visit. (b) Mouth opening of the patient (Case  1) after gap arthroplasty.

A 10-year-old girl, who suffered from limited mouth opening, was referred to our department in 2002 with a history of traffic accident one year earlier. Clinical examinations, conventional X-rays, and CT (Figure 1(a)) revealed Type 3 ankylosis [38] on the right TMJ. After explanation of the surgical procedures, the parents of the patient preferred autogenous grafts as interposition materials instead of any artificial material.

Under blind nasotracheal anesthesia, an endaural incision was used as described by Al-Kayat and Bramley [39]. Dissection was carried out through the superfacial temporal fossa, which was retracted anteriorly to protect the facial nerve, and the periosteum over the zygomatic arch was incised. To release ankylosis of the left TMJ, the subankylotic approach was preferred [40]. Bone was drilled with a round burs and two segments were split. A gap was created. Ankylotic bone was removed as much as possible from the upper segment. A satisfactory mouth opening (30 mm) was provided immediately after surgery (Figure 1(b)). Temporalis fascia was placed between two segments to prevent reankylosis.

After the operation, in order to improve and maintain interincisal opening (IO), the patient was urged to do vigorous exercises according to our treatment protocol [4].

**Figure 2:**
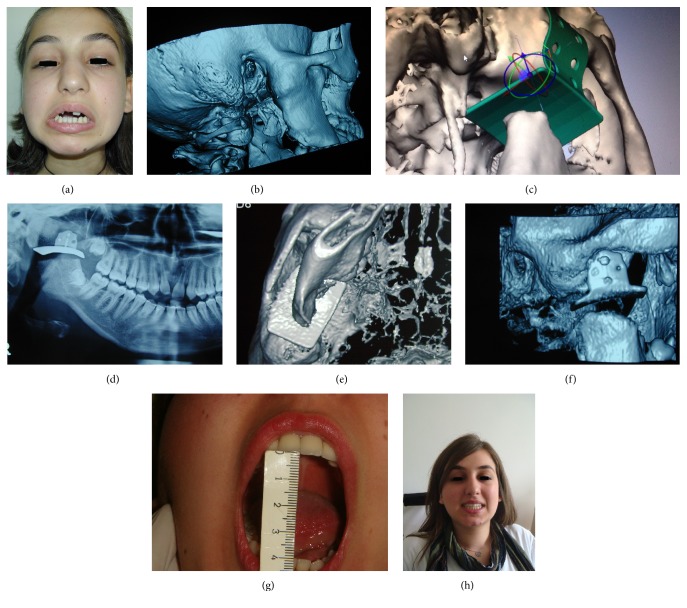
(a) The patient (Case  1) is not able to open her mouth due to reankylosis. (b) CT reveals reankylosis at the right TMJ five years after the first surgical treatment. (c, d) Panorex shows horizontal resection of the ramus via subankylotic approach. (e) The prosthesis creates a barrier to prevent reankylosis. (f) Complementation of the neoformation of the condyle two years after surgery. (g) Mouth opening, six years after surgical treatment. (h) Facial appearance after surgical and orthodontic treatment.

In 2007, the patient visited our department with the complaint of decreased mouth opening. Clinical examination (Figure 2(a)) and CTs revealed reankylosis; a thicker bony block was connected to the scull base (Figure 2(b)). In the second intervention, horizontal resection was made to release ankylosis as described before; after creating a gap, stock titanium fossa prosthesis (TFP)* (Confidence TMJ Hemijoint Prosthesis, Biotechnica Engineering, Medical Co. Ltd.)* was placed (Figures 2(c) and 2(d)) in order to prevent reankylosis [19]. Contralateral coronoidectomy was performed. The patient had the same postoperative care and physiotherapy as she previously had. In this second experience, the patient was apparently older and seemed to be determined to do painful exercises. One year after surgery, further clinical evaluation continued and 40 mm IO was achieved. X-ray was taken and remodeling of the resected ramus was clearly seen (Figure 2(e)). In the following years, clinical evaluations have continued. Two years after surgery, we found a pleasant surprise. A condyle-like process was observed resulting in a new form of ramus mandible (Figure 2(f)). After completion of orthodontic treatment, a satisfactory dentition and facial appearance was provided (Figure 2(g)). The patient was able to maintain 40 mm IO (Figure 2(h)) throughout the postoperative examination period of six years. Figure 2(e) shows how TFP creates a successful barrier and prevents the formation of heterotopic ossification and bony bridges between the two segments.

### 2.2. Case  2

**Figure 3:**
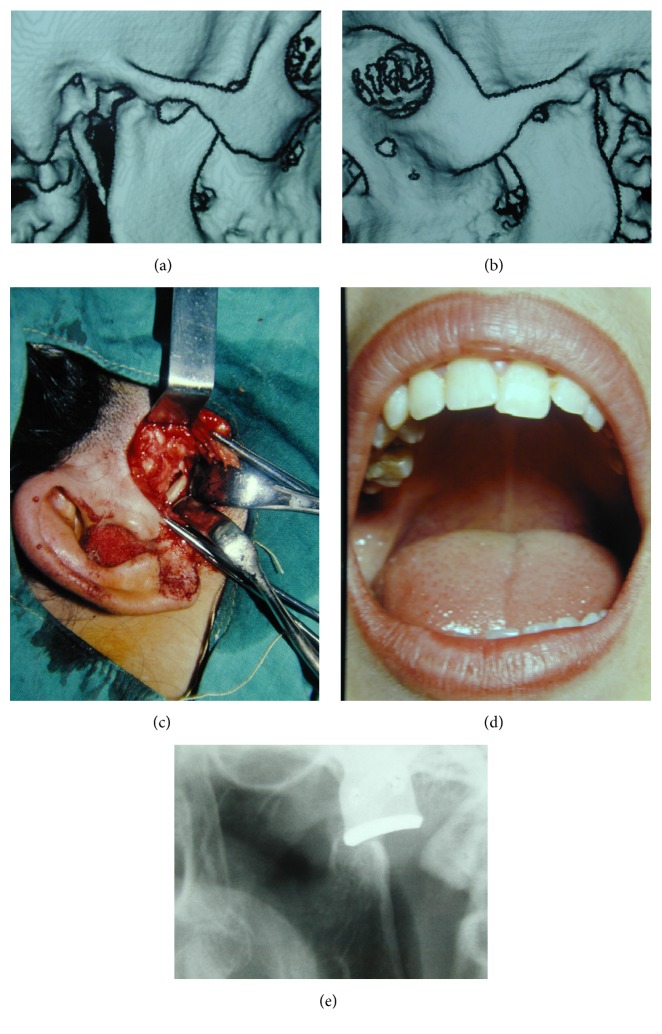
(a) During the examination of the patient (Case  2), CT revealed a spontaneously healed condyle after having a fracture at the right side. (b) Ankylosis at the left side (Case  2). (c) Horizontal resection of the ramus (Case  2). (d) Mouth opening provided by surgical treatment (Case  2). (e) Panorex shows a spontaneously healed condylar fracture at the right side and new condyle formation after surgical treatment of ankylosis at the left side (Case  2).

After falling from a height, a 23-year-old female patient was taken to a hospital and underwent a surgical operation for mandible fracture. Four months later, the patient experienced increasing difficulty in mastication and mouth opening. CTs revealed spontaneously healed condylar fracture at the right side (Figure 3(a)) and Type 2 ankylosis at the left side (Figure 3(b)). Under blind nasotracheal anesthesia, ankylotic bone was exposed, and burs horizontal resection was performed, and a satisfactory gap was created (Figure 3(c)). Eventually, free mandibular movement and 25 mm IO were attained. TFP was placed between the two segments and fixed by screws. The patient received physiotherapy immediately after surgery. In the following years, she had a satisfactory mouth opening (IO: 35 mm) (Figure 3(d)). X-rays regularly taken for seven years after surgery revealed the condyle shaped structure (Figure 3(e)).

## 3. Discussion

Ankylosis is a well known pathology of TMJ. Various techniques have been proposed for the treatment of this pathology, and yet none of them produced uniformly successful results. The first arthroplasty technique was tried by Percy and Burton [7] in 1826. They used muscle and fascia to prevent ankylosis. Although this technique was firstly proposed 188 years ago, it is still in use in some clinics.

In the following years, after releasing ankylosis, placing some prostheses to provide free movement of the mandible and to prevent reankylosis has been the primary goal for surgeons. All reports revealed postoperative IOs and contributed to the improvement of the technique the authors used. On the other hand, none of them reported any issue with regard to the changes in the resected segments in the long term.

Today, it is clear that it is possible to induce new condyle formation after condylar fracture by functional treatment in growing patients [2, 25, 26]. Similar to this phenomenon, some authors reported spontaneous bone regeneration in growing patients and in adults following resection of some proportions of the mandible [22, 29, 33, 35, 37, 41, 42].

Several suggestions have been made for spontaneous bone regeneration in the mandible. Fell [43] has shown that surface bone cells can survive and function in tissue culture. In resection of jaw bones, small particles of bone and in fact the “bone dust” may remain in the bed and exhibit osteogenic potential.

Urist et al. [44] explained how mesenchymal cells in connective tissue can be induced to form new bone. Growth factors play a major role in this process. The soft tissue surrounding the fracture site has been considered another contributor to fracture healing as a source of not only undifferentiated mesenchymal cells but also much-needed blood supply. Specifically, fracture hematoma has been found to contain the angiogenic cytokine vascular endothelial growth factor which has the inherent capability to induce angiogenesis and thus promote revascularization during bone repair [35, 45].

McKibbin [46] discussed the formation of primary callus that appeared as an initial reaction of bone to injury. Rapid widespread cellular activity that involves the surrounding soft tissues takes place in order to form a bridging external callus whose primary purpose is to maintain the stability of fragments. Once the bridge is formed, remodeling then proceeds to form mature bone from temporary callus. The periosteum is believed to be the primary source of the osteogenic tissue.

Einhorn [47] declared that the presence of committed and uncommitted undifferentiated mesenchymal cells in the periosteum contributes to the process of fracture healing by recapitulation of the embryogenic intramembranous and endochondral bone formation [35]. Kisner [31] reported that, for the cases in which the periosteum is not intact, the source of the regenerated bone could be fragments of periosteum, pieces of devitalized bone in the surrounding tissue, and the remaining mandibular stumps. It has been suggested that three conditions must be present for bone induction to occur: an inducing agent, an osteogenic precursor cell, and an environment which is permissive to osteogenesis [48]. In light of all these explanations with regard to new bone formation, the following question arises: Can this mechanism play a role in formation of reankylosis? It is possible; nevertheless, a satisfactory spacer and a successful postoperative physiotherapy prevent undesirable bone formation. Moreover, it may form a condyle-like structure. Figure 2(e) shows how the fossa prosthesis is an effective barrier in the prevention of reankylosis. It prevents formation of bony bridges between two segments.

There is another question of whether immobilization plays a role in promoting the regeneration process. While some authors [35, 37] stabilized the mandibular stump, others [22, 30] merely closed the wound and allowed the full range of motion of the mandible. Vigorous exercises started immediately after the surgery in both cases presented in our study. Shuker [41] declared that continuous functional stress on regenerating area could serve as a mechanical factor in promoting osteogenesis.

When soft tissues are prevented from collapsing into the mandibular defect by a rigid spacer, bone regeneration may be allowed to proceed unhindered as demonstrated by Boyne [32] and Güven and Tekin [49]. Following resection of the mandible for various types of neoplastic disease, six patients between the ages of 5 and 14 were examined regularly in the study of Boyne [32], for a period of 8 to 12 years after osseous surgical reconstructive surgery. It was found in children of this age group that a surgical technique may be employed which will effect complete bone regeneration without the use of bone graft materials. All six patients spontaneously regenerated large segments of the mandible from full-body ostectomy to hemimandibulectomy defects. This technique is advocated by Boyne [32], for bone restoration in cases of large osseous discontinuity defects of the mandible in children.

A limited number of older patients with spontaneous healing have been reported so far. The patient reported by Budal [29] was 35 years old, the patient reported by Elbeshir [42] was 32 years old, the patient reported by de Villa et al. [35] was 58 years old, and one of the cases presented in our report was 23 years old. This shows that this phenomenon is not always limited by age but can remain potent throughout the lifetime of an individual and may be induced under certain circumstances [35].

In most of the reported cases [22, 27, 32, 34–36, 41, 42], mandibular body or ramus showed rapid bone regeneration or spontaneous healing. Few of them, Nagase et al. [33] and Khodayari et al. [37], reported the regeneration of the condyle following hemimandibulectomy (Table 1). Nagase et al. [33], and Khodayari et al. [37] noted the importance of the preservation of periosteum and declared that the immediate reconstruction of defects with autogenous bone that occur in children or adolescents is an issue still open to discussion.

Historically, the condyle has been regarded as a kind of cornucopia from which the whole mandible pours forth. On the other hand, present-day biology scholars do not regard the condyle as a structure merely functioning to regulate morphogenesis of the whole mandible [50]. Enlow and Hans [50] reported that, with regard to the growth and adaptive requirements of the mandible, not only the condyle but also the whole ramus is directly involved in the process. In essence, the condyle follows the growth of the whole ramus and does not lead it. In light of the suggestions of Enlow and Hans [50], in some of the patients, should we expect to have condylar reformation induced by ramus mandible after the resection of the TMJ ankylosis? We observed this formation in two of our patients. In the first presented case, one year after second surgery, besides having a satisfactory mouth opening and free mandibular movements, X-ray revealed a remodeling on the mandibular stump and in the following year a bony structure resembling condyle was clearly observed (Figure 2(f)). Within the 2nd year after surgery, the X-rays of the second patient revealed neocondyle formation (Figure 3(e)). According to the treatment protocol [1], after creating a gap, resected mandibles were not shaved by the burs. Only TFP was placed.

## 4. Conclusion

In patients with TMJ ankylosis, the ramus connects to the base and naturally we cannot see a condyle at the posterosuperior part of the ramus. Despite using the same surgical technique to relieve ankylosis, only two patients had unexpected formation. The condyle-like process formation in the presented two cases brings some questions: Which conditions lead to this formation? Another question of interest is why do some individuals regenerate new bone so rapidly and spontaneously while others do not? The biological reasons and/or genetic factors may require further investigation [22]. The following three mechanisms may account for the condyle-like bone formation in that two patients we dealt with:

1. The mechanisms of fracture healing with growth factors provide stimulus, and the surrounding soft tissues provide nourishment for undifferentiated mesenchymal cells for a new osteogenic tissue. However, it is still unclear which factors or combinations thereof favor this process [35].
2. The polished, concave surface of the prosthesis [24] provides free movement of the resected segment and this leads to remodeling of the ramus and formation of a condyle-like structure.
3. Self-discipline of the patient during physiotherapy is a very important parameter to prevent reankylosis, which causes bone deposition and formation of condyle-like process.

The possibility of lack of growth or overgrowth in reconstruction of jaws by autogenous grafts and advantages and disadvantages of reconstruction of the defects by artificial materials such as total joint prostheses have been discussed previously. The necessity of placing reconstructive materials or total joint prostheses in growing patients is still a matter of controversy. Further studies which will enable physicians to understand and control the mechanisms of spontaneous bone formation in jaws will give some opportunities to treat patients, particularly growing ones, without using artificial materials.

## Figures and Tables

**Table 1:** Cases of spontaneous condylar reformation reported in the English literature.

Number of cases	Author	Age	Gender	Diagnosis	Treatment
1	Nagase et al., 1985	12	M	Ameloblastoma	Partial mandibulectomy
1	Khodayari et al., 2011	19	M	Keratocyst	Partial mandibulectomy

## References

[1] Tanrikulu R., Erol B., Görgün B., Söker M. (2005). The contribution to success of various methods of treatment of temporomandibular joint ankylosis (a statistical study containing 24 cases). *Turkish Journal of Pediatrics*.

[2] Güven O., Keskin A. (2001). Remodelling following condylar fractures in children. *Journal of Cranio-Maxillofacial Surgery*.

[3] Shang H., Xue Y., Liu Y., Zhao J., He L. (2012). Modified internal mandibular distraction osteogenesis in the treatment of micrognathia secondary to temporomandibular joint ankylosis: 4-Year follow-up of a case. *Journal of Cranio-Maxillofacial Surgery*.

[4] Güven O. (2000). A clinical study on temporomandibular joint ankylosis. *Auris Nasus Larynx*.

[5] Güven O. (2004). Treatment of temporomandibular joint ankylosis by a modified fossa prosthesis. *Journal of Cranio-Maxillofacial Surgery*.

[6] Gundlach K. K. H. (2010). Ankylosis of the temporomandibular joint. *Journal of Cranio-Maxillofacial Surgery*.

[7] Verneuil A. (1860). De la création d’une fausse articulation par section ou résection partielle de l’os maxillaire inférieure comme moyen de remédier a l2 ankylose vraie ou fausse de la mâchoire inférieure. *Archives General De Medecine*.

[8] Topazian R. G. (1966). Comparison of gap and interposition arthroplasty in the treatment of temporomandibular joint ankylosis. *Journal of oral Surgery*.

[9] Poswillo D. (1974). Experimental reconstruction of the mandibular joint. *International Journal of Oral Surgery*.

[10] Narang R., Dixon R. A. (1975). Temporomandibular joint arthroplasty with fascia lata. *Oral Surgery, Oral Medicine, Oral Pathology*.

[11] Cobey M. C. (1967). Arthroplasties using compressed ivalon sponge ("intra-medic sponge") long-term follow-up studies in 109 cases. *Clinical Orthopaedics and Related Research*.

[12] Borçbakan C. (1968). L’utilisation du condyle acrylique dans l’ankylose temporo-maxillaire. *Revue de Stomatologie et de Chirurgie Maxillo-Faciale*.

[13] DeChamplain R. W., Gallagher C. S., Marshall E. T. (1988). Autopolymerizing silastic for interpositional arthroplasty. *Journal of Oral and Maxillofacial Surgery*.

[14] Cope M. R., Moos K. F., Hammersley N. (1993). The compressible silicone rubber prosthesis in temporomandibular joint disease. *British Journal of Oral and Maxillofacial Surgery*.

[15] Christensen R. W., Cranin M. N. (1971). Arthroplastic implantation of the temporomandibular joint. *Oral Implantology*.

[16] Kent J. N., Misiek D. J., Akin R. K., Hinds E. C., Homsy C. A. (1983). Temporomandibular joint condylar prosthesis: A ten-year report. *Journal of Oral and Maxillofacial Surgery*.

[17] Wolford L. M., Cottrell D. A., Henry C. H. (1994). Temporomandibular joint reconstruction of the complex patient with the techmedica custom-made total joint prosthesis. *Journal of Oral and Maxillofacial Surgery*.

[18] Mercuri L. G. (2000). The use of alloplastic prostheses for temporomandibular joint reconstruction. *Journal of Oral and Maxillofacial Surgery*.

[19] Güven O. (2008). A clinical study on temporomandibular joint ankylosis in children. *Journal of Craniofacial Surgery*.

[20] Keller E. E., Baltali E., Liang X., Zhao K., Huebner M., An K.-N. (2012). Temporomandibular custom hemijoint replacement prosthesis: Prospective clinical and kinematic study. *Journal of Oral and Maxillofacial Surgery*.

[21] Tschopp H. M., Spiessl B. (1976). Clinical aspects of free autogenous bone transplantation. *New Concepts in Maxillofacial Bone Surgery*.

[22] Nwoku A. L. (1980). Unusually rapid bone regeneration following mandibular resection. *Journal of Maxillofacial Surgery*.

[23] Davis P. K. B., Jones S. M. (1971). The complications of silastic implants. Experience with 137 consecutive cases. *British Journal of Plastic Surgery*.

[24] Güven O. (2010). Bidirectional temporomandibular joint ankylosis: A rare, disabling condition of mastication. *Journal of Craniofacial Surgery*.

[25] Baume L. J., Derichsweiler H. (1961). Response of condylar growth cartilage to induced stresses. *Science*.

[26] Waite D. E. (1973). Pediatric fractures of jaw and facial bones. *Pediatrics*.

[27] Kazanjian V. H. (1946). Spontaneous regeneration of bone following excision of section of the mandible. *American Journal of Orthodontics and Oral Surgery*.

[28] Byars L. T., Schatten W. E. (1960). Subperiosteal segmental resection of the mandible. *Plastic and Reconstructive Surgery*.

[29] Budal J. (1970). The surgical removal of large osteofibromas. The postoperative osteogenic capacity of the periosteum. *Oral Surgery, Oral Medicine, Oral Pathology*.

[30] Adekeye E. O. (1977). Rapid bone regeneration subsequent to subtotal mandibulectomy. Report of an unusual case. *Oral Surgery, Oral Medicine, Oral Pathology*.

[31] Kisner W. H. (1980). Spontaneous posttraumatic mandibular regeneration. *Plastic and Reconstructive Surgery*.

[32] Boyne P. J. (1983). The restoration of resected mandibles in children without the use of bone grafts. *Head & Neck Surgery*.

[33] Nagase M., Ueda K., Suzuki I., Nakajima T. (1985). Spontaneous regeneration of the condyle following hemimandibulectomy by disarticulation. *Journal of Oral and Maxillofacial Surgery*.

[34] Whitmyer C. C., Esposito S. J., Smith J. D., Zins J. E. (1979). Spontaneous regeneration of a resected mandible in a preadolescent: A clinical report. *Journal of Prosthetic Dentistry*.

[35] de Villa G. H., Chen C. T., Chen Y. R. (2003). Spontaneous bone regeneration of the mandible in an eldery patient: A case report an review of the literature. *Chang Gung Medical Journal*.

[36] Pramono C. D. (2004). Spontaneous bone regeneration after mandible resection in a case of ameloblastoma - A case report. *Annals of the Academy of Medicine Singapore*.

[37] Khodayari A., Khojasteh A., Kiani M. T., Nayebi A., Mehrdad L., Vahdatinia M. (2011). Spontaneous regeneration of mandible after hemimandibulectomy: Report of case. *Journal of Dentistry Tehran University Medical Sciences*.

[38] Sawhney C. P. (1986). Bony ankylosis of the temporomandibular joint: Follow-up of 70 patients treated with arthroplasty and acrylic spacer interposition. *Plastic and Reconstructive Surgery*.

[39] Al-Kayat A., Bramley P. (1979). A modified pre-auricular approach to the temporomandibular joint and malar arch. *British Journal of Oral Surgery*.

[40] Salins P. C. (2000). New perspectives in the management of craniomandibular ankylosis. *International Journal of Oral and Maxillofacial Surgery*.

[41] Shuker S. (1985). Spontaneous regeneration of the mandible in a child a sequel to partial avulsion as a result of a war injury. *Journal of Maxillofacial Surgery*.

[42] Elbeshir E. I. (1990). Spontaneous regeneration of the mandibular bone following hemimandibulectomy. *British Journal of Oral and Maxillofacial Surgery*.

[43] Fell H. B., Bourne G. H. (1956). Skeletal development in tissue culture. *The Biochemistry and Physiology of Bone*.

[44] Urist M. R., Hay P. H., Dubuc F., Buring K. (1969). Osteogenic competence. *Journal of Clinical Orthopaedics*.

[45] Street J., Winter D., Wang J. H., Wakai A., McGuinness A., Redmond H. P. (2000). Is human fracture hematoma inherently angiogenic?. *Clinical Orthopaedics and Related Research*.

[46] McKibbin B. (1978). The biology of fracture healing in long bones. *Journal of Bone and Joint Surgery B*.

[47] Einhorn T. A. (1998). The cell and molecular biology of fracture healing. *Clinical Orthopaedics & Related Research*.

[48] Chalmers J., Gray D. H., Rush J. (1975). Observations on the induction of bone in soft tissues. *The Journal of Bone & Joint Surgery—British Volume*.

[49] Güven O., Tekin U. S. (2006). Healing of bone defects by an osteopromotion technique using solvent-dehydrated cortical bone plate: A clinical and radiological study. *Journal of Craniofacial Surgery*.

[50] Enlow D. H., Hans M. G. (1996). *Essentials of Facial Growth*.

